# Environmentally Driven Color Variation in the Pearl Oyster *Pinctada margaritifera* var. *cumingii* (Linnaeus, 1758) Is Associated With Differential Methylation of CpGs in Pigment- and Biomineralization-Related Genes

**DOI:** 10.3389/fgene.2021.630290

**Published:** 2021-03-19

**Authors:** Pierre-Louis Stenger, Chin-Long Ky, Céline M. O. Reisser, Céline Cosseau, Christoph Grunau, Mickaël Mege, Serge Planes, Jeremie Vidal-Dupiol

**Affiliations:** ^1^IFREMER, UMR 241 Écosystèmes Insulaires Océaniens, Labex Corail, Centre du Pacifique, Tahiti, French Polynesia; ^2^IHPE, Université de Montpellier, CNRS, IFREMER, Université de Perpignan Via Domitia, Montpellier, France; ^3^MARBEC, Université de Montpellier, CNRS, IFREMER, IRD, Montpellier, France; ^4^IHPE, Université de Montpellier, CNRS, IFREMER, Université de Perpignan Via Domitia, Perpignan, France; ^5^IFREMER, PDG-RBE-SGMM-LGPMM, La Tremblade, France; ^6^EPHE-UPVD-CNRS, USR 3278 CRIOBE, Labex Corail, PSL Research University, Université de Perpignan, Perpignan, France

**Keywords:** pearl oyster, environmental pressure, depth, color change, pigmentation, DNA methylation, methylome characterization

## Abstract

Today, it is common knowledge that environmental factors can change the color of many animals. Studies have shown that the molecular mechanisms underlying such modifications could involve epigenetic factors. Since 2013, the pearl oyster *Pinctada margaritifera* var. *cumingii* has become a biological model for questions on color expression and variation in Mollusca. A previous study reported color plasticity in response to water depth variation, specifically a general darkening of the nacre color at greater depth. However, the molecular mechanisms behind this plasticity are still unknown. In this paper, we investigate the possible implication of epigenetic factors controlling shell color variation through a depth variation experiment associated with a DNA methylation study performed at the whole genome level with a constant genetic background. Our results revealed six genes presenting differentially methylated CpGs in response to the environmental change, among which four are linked to pigmentation processes or regulations (*GART*, *ABCC1*, *MAPKAP1*, and *GRL101*), especially those leading to darker phenotypes. Interestingly, the genes *perlucin* and *MGAT1*, both involved in the biomineralization process (deposition of aragonite and calcite crystals), also showed differential methylation, suggesting that a possible difference in the physical/spatial organization of the crystals could cause darkening (iridescence or transparency modification of the biomineral). These findings are of great interest for the pearl production industry, since wholly black pearls and their opposite, the palest pearls, command a higher value on several markets. They also open the route of epigenetic improvement as a new means for pearl production improvement.

## Introduction

Pearls have captivated and amazed human civilization since 7500 BP (Charpentier et al., 2012) and have formed the basis for economic development of several tropical countries. French Polynesia started to trade pearls with Europeans at the end of the 18th century (Southgate and Lucas, 2011) and has been an industrial producer since 1964 (Le Pennec, 2010). Today, cultured pearl farming represents the second economic resource of the country, just behind tourism (Ky et al., 2019). However, since 2001, an unprecedented economic crisis has endangered the French Polynesian pearl farming sector due to the overproduction of low quality pearls (Bouzerand, 2018). To remediate to this socio-economic crisis, the policy of producing “less but better” was adopted in 2013 (Ky et al., 2013). Since then, production has focused on obtaining cultured pearls with high market value, by selecting traits such as pearl color.

Two individuals are needed to produce a pearl, a donor and a recipient oyster. The sacrificed donor is used to provide a piece of mantle (the biomineralizing graft) which is placed, together with a small marble of nacre, into the gonad of the recipient oyster. Donor oysters are selected for their inner-shell color, since the color of a cultured pearl is determined by that of the donor oyster (Ky et al., 2013). Recipient oysters are selected for their vigor. The Polynesian black-lipped pearl oyster, *Pinctada margaritifera* var. *cumingii* (Linnaeus, 1758) is the pearl oyster species showing the largest range of inner shell color (Ky et al., 2013, 2017), therefore offering a wide range of pearl colors and shades, like dark, pastel, silver, peacock, red, golden, green, blue, and even rainbow (Ky et al., 2014; Stenger et al., 2019). Moreover, the color of this bivalve has both a structural (iridescence; Liu et al., 1999) and a biological (pigments; Iwahashi and Akamatsu, 1994) basis. While the genetic determination of this color has been partly demonstrated (Ky et al., 2013, 2017), several environmental factors are also known to affect the shell color (Joubert et al., 2014; Le Pabic et al., 2016; Le Moullac et al., 2018), such as the depth at which an oyster is grown (Stenger et al., 2019). In this latter work, authors demonstrated that the transplantation of oysters from the sub-surface (−4 m) to the bottom of the lagoon (−30 m) significantly darkens the inner shell color compared with oysters maintained at the sub-surface. This induced phenotype was persistent through time (“enduring”), even after the oysters were returned to shallow water, which would seem to indicate an epigenetic control mechanism rather than a direct environmental influence acting on the darkening of the shell color (Stenger et al., 2019).

Since the first definition of epigenetic by Waddington in the late 1930s, epigenetic received many definitions (Nicoglou and Merlin, 2017). In this work we have selected the definition proposed by Russo et al. (1996), e.g., *“epigenetic is the study of mitotically and/or meiotically heritable changes in gene function that cannot be explained by changes in DNA sequence”* (Russo et al., 1996). Mechanistically, this memory function is based on changes in chromatin structure, such as non-coding RNA and/or histone modifications and/or DNA methylation. Here, we use the term epigenetics to describe any changes in DNA methylation that occur upon environmental cues. Coloration mediates an organism’s relationship with their environment in important ways including anti-predator defenses, social signaling, thermoregulation, or protection (Cuthill et al., 2017). Several species are known to change their coloration more than once in their lifetime in response to environmental triggers to reach an optimal phenotype in a new environment. To increase their camouflage the arctic hare *Lepus arcticus* (Ross, 1819), the ermine *Mustela erminea* (Linnaeus, 1758), and the ptarmigan *Lagopus muta* (Montin, 1776) changed their coat color from brown or gray in the summer to white in the winter (Zimova et al., 2018). These changes are known to be induced by temperature, photoperiod, and/or food rarefaction (Zimova et al., 2018). The corresponding changes in color expression could be brought by epigenetic processes (Hu and Barrett, 2017) like in mouses (Dolinoy, 2008). Indeed, the most striking example of color change in mammals rely on the environmentally induced differential DNA methylation of the intracisternal A-particle gene located upstream of the agouti locus (Dolinoy et al., 2006; Dolinoy, 2008). In Mollusca, a few epigenetic studies have been made, especially on color expression and variation. To date, Feng et al. (2018) provided a catalog of long non-coding RNA (lncRNA) expressed in the mantle of the Pacific oyster *Crassostrea gigas* (Thunberg, 1793). These authors suggested that these lncRNAs may affect the expression of pigment-related genes such as tyrosinase-like proteins, dopamine, beta-monooxygenase, chorion peroxidase, or cytochrome P450 2U1, thus leading to different shell color phenotypes. More recently, the same group (Feng et al., 2020) have studied the role of microRNAs in the regulation of the shell color of *Crassostrea gigas*. In this study, four miRNAs (lgi-miR-315, lgi-miR-96b, lgi-miR-317, and lgi-miR-153) were found closely associated with shell color along with the regulation of Cytochrome P450 2U1, Tyrosinase-like protein 2 and 3. The authors concluded that lgi-miR-317, its targeted mRNA encoding peroxidase, and the lncRNA TCONS_00951105 might play a key role in shell melanin synthesis.

Because color phenotype is important for the pearl market and the phenotypic plasticity of this trait is associated with the putative involvement of epigenetic mechanisms, the study of these mechanisms opens a new avenue for improvement in the pearl industry with, for example, the development of epi-markers for environmentally induced color variation testing. As a first step in exploring a possible interaction between epigenetic mechanisms and color variation, we designed a depth variation experiment to induce environmentally driven color variation. In order to disentangle the genetic factors from the epigenetic ones influencing color variation, a non-lethal sampling design was used enabling us to monitor changes in DNA methylation over time and depth within the same individuals (constant genotypes). DNA methylation was studied at the whole genome scale by whole-genome bisulfite sequencing and provided evidence for an epigenetic control of pearl oyster color variation. This approach enabled us to find any differences in DNA methylation in pearl oysters after a period at increased depth and, when this occurred, to examine whether genes related to pigmentation and/or biomineralization processes were affected by such changes. Results of this kind could allow the pearl industry to turn to more sustainable production strategies.

## Materials and Methods

### Biological Material and the Yo-Yo Experiment

**FIGURE 1:**
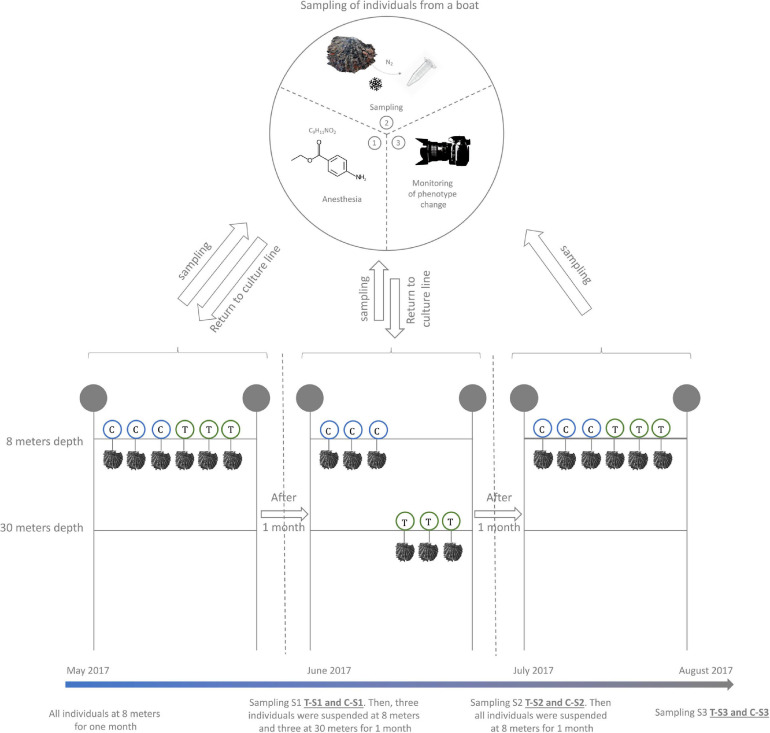
Design of the Yo-Yo experiment. Six *P. margaritifera* individuals were used for this experiment. Three individuals were maintained at a depth of 8 m during the whole experiment and were used as a control (C – in blue circles). The three other individuals were subjected to depth variation treatment (T – in green circles). A non-lethal sampling of mantle tissue was made every month at the time of the depth change.

In order to trigger an environmentally driven color change of the inner shell of *P. margaritifera*, we set up an *in situ* “yo-yo” experiment (May to August 2017) (Figure 1). Six individuals of 4 years of age (approximately 14 cm height) originating from three different families (two individuals per family Stenger et al., in press) were used. These six individuals were first maintained at 8 m depth for 1 month (May 2017). Then, three of them (1 from each family) were selected and transferred to 30 m depth (treatment) for environmental pressure while the three others were left at 8 m (control). This exposure was maintained for 1 month (June 2017). Then, the three pearl oysters that had been placed at 30 m were transferred back to 8 m depth for a final month of exposure (July 2017). During each transfer, a piece of mantle (the biomineralizing tissue responsible for the inner shell coloration) was sampled by a non-lethal method: (i) oysters were anesthetized in 20 L seawater containing 200 mL benzocaine at 120 g/L 96° ethanol under air aeration; (ii) they opened their valves under the effect of the benzocaine, a 5 mm^3^ fragment of the mantle was carefully sampled with tweezers and scissors; (iii) the sample was flash frozen in liquid nitrogen. Alongside the sampling of the mantle, the color of the inner shell of each individual (control and treatment) was filmed with a mini photo studio for color variation analysis. This mini standardized photo studio was composed of a tripod supporting a Nikon D3100 reflex camera equipped with a Nikon 18-55VR lens. This set up was used to film the reflection of the inner shell color on a small mirror (spatula). To assess constant exposition to light, all films were made under a blackout drape with three white LED lamps.

### Color Variation Analysis

For each sample, ten screenshots from the films were randomly captured and analyzed for color variation. Color was quantified and qualified using the R package *ImaginR* V.2 (Stenger, 2017) as previously described (Stenger et al., 2019). A Shapiro test (stats v3.5.0 R package) was used to assess the normal distribution of the data. The average saturation and darkness for each sample were calculated from all ten screenshots, and a Wilcoxon test (*stats v3.5.0* R package based on Hollander et al., 1973 and Royston, 1995) was used to test for any difference between the groups.

### DNA Extraction and BS-seq

DNA was extracted with the QIAamp DNA Mini Kit (QIAGEN^®^, Cat No./ID: 51306) following manufacturer’s recommendations, with the addition of two steps: (i) a RNase A treatment to remove RNA and (ii) after overnight digestion the addition of 50 μL of a saturated KCl solution (34 g KCl/100 g H_2_O) and a centrifugation at 14,000*g* for 15 min to remove the mucopolysaccharides (Sokolov, 2000). DNA quality and quantity were assessed with a NanoDrop^TM^ 2000 and 1.2% agarose gel electrophoresis. Bisulfite-conversion, library construction, and sequencing (2 × 150 bp) were performed by Genome Québec (MPS Canada) on an Illumina HiSeq X.

### Bioinformatics Pipeline

Analyses were performed on the Ifremer Datarmor cluster^1^. Raw read quality was assessed with FastQC (Andrews, 2010; Supplementary Table 1). Reads were cleaned and adaptors removed with Trimmomatic (Bolger et al., 2014) (V. 0.36 – illuminaclip 2:30:10; leading 28, trailing 28, and minlen 40). Bismark aligner (Krueger and Andrews, 2011) (V. 0.19) was used to map reads on the draft genome of *P. margaritifera* (Reisser et al., 2020) using the following parameters: multi-seed length of 30 bp, 0 mismatches, default minimum alignment score function. Deduplication was done with Deduplicate_bismark (Krueger and Andrews, 2011). Bed files for methylome characterization were obtained with Bismark_methylation_extractor (Krueger and Andrews, 2011). All scripts are provided on GitHub (PLStenger/Pearl_Oyster_Colour_BS_Seq/00_scripts). Raw reads are available through the NCBI Sequence Read Archive (SRA, BioProject PRJNA663978, BioSample SAMN16191417 to SAMN16191446).

### Methylation Calling, Methylome Characterization, and Differential Methylation Analysis

The R package *Methylkit* (Akalin et al., 2012) (V. 1.11.0) was used for methylation calling in CpG, CHH, and CHG contexts using a minimum coverage of 10, directly from BAM files with *processBismarkAln*. *Methylkit* was also used for methylation characterization in the CpG, CHH, and CHG contexts, as well as for the coverage calculation and clustering analysis (ward clustering correlation distance method). The average gene methylation was calculated with DeepTools V. 3.3.0 (Ramírez et al., 2014). The gene body methylation rate (GBMR), corresponding to the CpG methylation rate, was calculated with the map function from bedtools V. 2.27.1 (Quinlan and Hall, 2010).

The mantle’ gene expression data used for methylation/gene expression correlation analysis came from individuals studied in Stenger et al., in press (SRA BioProject PRJNA521849). Briefly, RNA sequencing was done using high quality RNA extracted from twelve individuals. Library construction and sequencing was performed (Paired–end 100-bp; Illumina^®^ HiSeq^®^ 4000) by Génome Québec (MPS Canada). Read quality check and trimming were done as described in the section “Bioinformatics Pipeline.” Cleaned reads were paired-mapped against the *P. margaritifera* draft genome with TopHat (V1.4.0) (Trapnell et al., 2012). Cufflinks (V2.2.1.0) and Cuffmerge (V2.2.1.0) were used to assemble and merge the transcriptome produced for each library, respectively (Trapnell et al., 2012). HTSeq-count (V0.6.1) (Anders et al., 2015) was used to count read-mapped per transcript. The average of the twelve count files was computed and the RPKM method was used for gene expression data normalization as previously done in Wang et al., 2014.

To identify the effect of the depth treatment, differentially methylated CpGs (DMCpGs) were identified with the *getMethylDiff* function of the R package *Methylkit*, with difference >25% and *q* value < 0.05 for CpG positions between depth treatment and control oysters at each sampling time.

### Functional Analysis of Differentially Methylated Genes

An annotation file was obtained following the first three steps of https://github.com/enormandeau/go_enrichment, completed with PLASTX (Nguyen and Lavenier, 2009) against UniProt-Swiss-Prot and TrEMBL (*e*-value at 1 × 10^–3^). A protein domain search was then performed with InterProScan. Finally, Gene Ontology terms were assigned with Blast2GO by combining information from the two annotation files (Conesa et al., 2005).

GOATOOLS (Klopfenstein et al., 2018) was used to test for enrichment of GO terms in a selected set of genes (significantly differentially methylated, lowly and highly methylated), using a Fisher’s exact test. Histograms showing the GO terms enrichment of were generated with the *ggplot2* R package (Wickham and Chang, 2019) (V. 2.2.1).

## Results

### Depth Variation Induces Significant Darkening

The color variation analysis with the *ImaginR* package detected no significant modification of hue, saturation or darkness among the controls during the 3 months of the “yo-yo” experiment (pairwise Wilcoxon tests with *P* values > 0.05). Among the samples subjected to the treatment, no significant change occurred for hue or saturation during the 3 months of the experiment. However, a significant difference in darkness (pairwise Wilcoxon tests with *P* values < 1.10e^–5^) was detected, with an increase in the darkness value during the month at 30 m depth. This increase was still visible and significant after the oysters were returned to 8 m depth for 1 month (pairwise Wilcoxon tests with *P* values < 1.10e^–5^; Table 1). Raw data are available for download using the following links (screenshots: https://figshare.com/articles/dataset/ImaginR_raw_data_screenshots_zip/14049983; videos: https://figshare.com/articles/dataset/ImaginR_raw_data_videos_zip/14049986).

**TABLE 1:** Darkness values obtained with the *ImaginR* R package analysis.

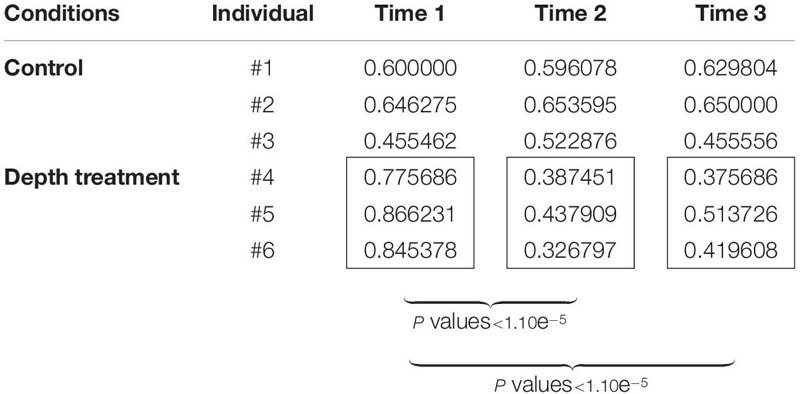

Only *P. margaritifera* individuals from the depth treatment group showed significant differences (pairwise Wilcoxon tests with *P* values < 1.10e^–5^ ***) between sampling times 1 and 2, and sampling times 1 and 3. Sampling times 1, 2, and 3 correspond to June, July and August 2017, respectively, as explained in Figure 1.

### Read Processing and Read Mapping to the Reference Genome

For the control, Illumina sequencing produced averages of 105,505,318 (±1,514,514; *n* = 3), 103,770,226 (±5,087,254; *n* = 3), and 103,490,158 (±5,509,306; *n* = 3) raw sequence reads for sampling times 1, 2, and 3, respectively. For the depth treatment, averages of 98,184,541 (±6,186,502; *n* = 3), 104,727,236 (±6,141,350; *n* = 3), and 113,132,278 (±4,306,828; *n* = 3) raw sequence reads were produced for sampling times 1, 2, and 3, respectively. After cleaning and filtering, averages of 101,575,689 (±1,584,202), 100,159,677 (±5,076,306), and 100,073,964 (±5,415,409) reads were kept for the control, and 94,669,211 (±5,877,099), 101,048,705 (±6,094,775), and 109,246,931 (±4,195,331) for the depth treatment, for the three successive sampling times, respectively. Filtered reads were mapped on the reference genome with Bismark and showed similar mapping rates for all samples (∼32.1%) (Supplementary Table 2). PCR duplicates were removed and represented, on average, 0.059% (±0.002) of the total reads.

### Characterization of the *P. margaritifera* Mantle Methylome

**FIGURE 2:**
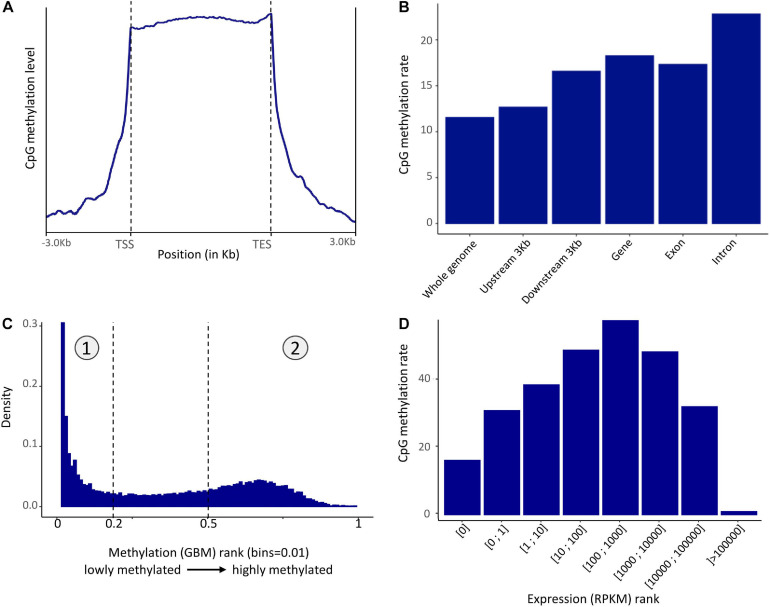
**(A)** Pattern of methylation level in the mantle of *P. margaritifera* (TSS, Transcription Start Site; TES, Transcription End Site) reported on a metagene. **(B)** Distribution of methylated CpG in different genomic regions. **(C)** Distribution of methylated CpG across genes ranked according to methylation level. The left part (1) is composed of lowly methylated genes while the right part (2) is composed of highly methylated genes. **(D)** CpG methylation rate averaged across rank of gene expression.

Methylation in the *P. margaritifera* mantle displayed a mosaic pattern with an enrichment in the gene showing a CpG methylation rate of 22.81% in introns, 17.32% in exons, and an average of 18.26% in gene body (Figures 2A,B). A slight enrichment in the 3 kbps upstream (12.67%) and downstream (16.58%) of genes (Figures 2A,B) is also found. The whole genome CpG methylation rate is equal to 11.53% on average.

The fractionation of gene body methylation rate (Figure 2C) shows a bimodal distribution characterized by two peaks. This distribution enables the classification of genes into two sets: the “lowly” methylated genes (0.00–0.19% methylation – rank 0–0.2 and marked as “1” Figure 2C) and the “highly” methylated genes (0.50–40.12% of methylation; 40.12% is the maximum methylation found in a gene in these data – rank 0.5–1 and marked as “2” Figure 2C). The enrichment analysis performed on the lowly methylated genes showed an overrepresentation of GO terms involved in reproductive process functions, such as “oocyte maturation” (GO:0001556), “primary sex determination” (GO:0007539), “male meiosis I” (GO:0007141), etc., cellular signaling, such as “negative regulation or cellular response to drug” (GO:0048523), “negative regulation of phospholipid metabolic process” (GO:0071072), etc., and “androgen receptor signaling pathway” (GO:0030521). In the highly methylated genes set, the enrichment analysis showed an overrepresentation of GO terms involved in housekeeping functions, such as “mRNA regulation” (GO:0043488), “mRNA splice site regulation” (GO:0006376), “ribosomal small unit assembly” (GO:0000028), “transcription-dependent tethering of RNA polymerase II” (GO:0000972), “DNA amplification” (GO:0006277), etc (Supplementary Figure 1).

To test for a correlation between the GBMR and the level of gene expression, the distribution of gene body methylation levels was represented according to gene expression rank (in RPKM). This distribution revealed that moderately expressed genes (100–1000 RPKM) have higher methylation levels than lowly (>100 RPKM) or highly (<1000) expressed ones in *P. margaritifera* (Figure 2D).

### Methylation Calling and Differential Methylation Analysis

**FIGURE 3:**
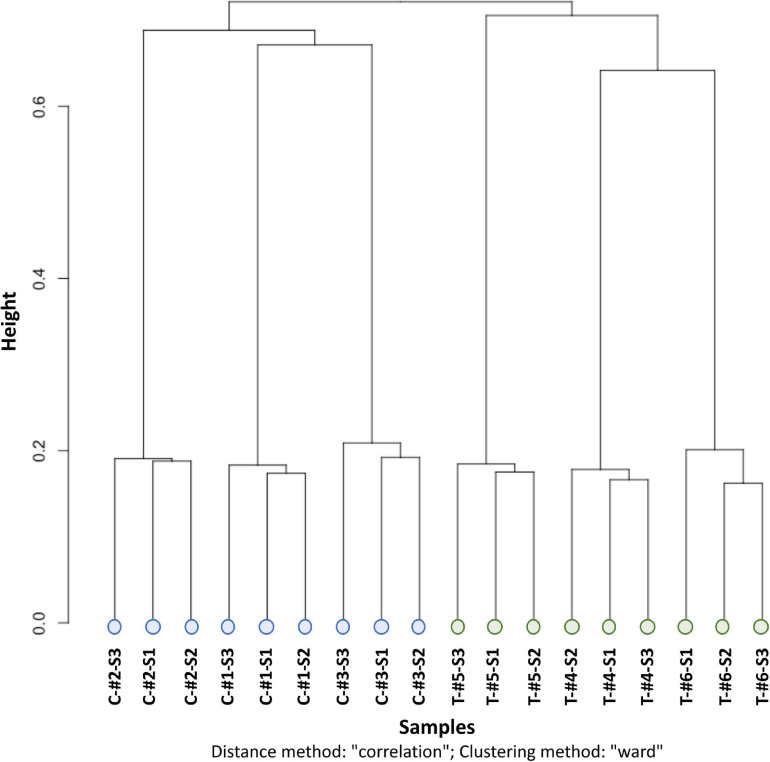
CpG methylation clustering with the distance method “correlation” and clustering method “ward” from the clusterSamples function of the R package *Methylkit*. C, control individual (in blue); T, depth treatment individual (in green); #, label of the individual number; S1, sampling time 1; S2, sampling time 2; S3, sampling time 3.

Methylation calling showed that the Cytosine methylation level was in average of 16.5% [12.0% in the CpG context, 0.9% in the CHG context, 1.0% in the CHH context, and 2.6% in another context (CN or CHN); Supplementary Figure 2]. Similarities between the methylation patterns of each sample were analyzed by a clustering approach (ward clustering correlation distance method). This showed that samples clustered first by treatment (depth vs. control), then by genotype (i.e., individuals) and, for the control only, by sampling time (Figure 3).

**FIGURE 4:**
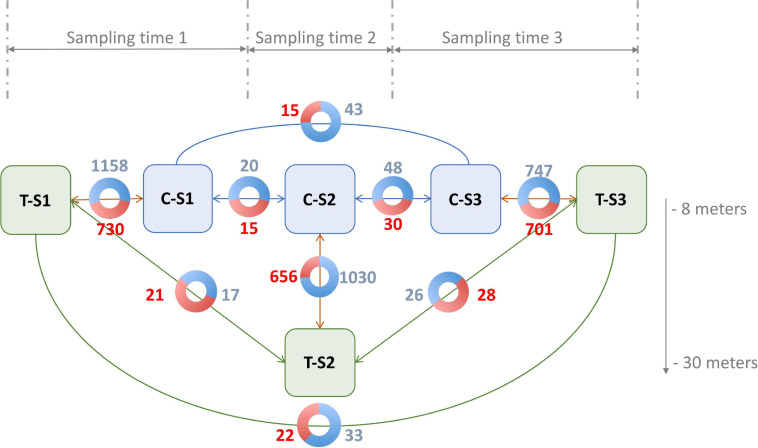
Number of significant hypermethylated (in red) and hypomethylated (in blue) positions (*q* < 0.05) in the nine pairwise comparisons. Blue boxes are the control groups, green boxes are the treatment groups. T, depth treatment individuals (in green); C, control individuals (in blue); S, sampling time.

For the differential methylation analysis, triplicates were made according to treatment (depth and control), and nine pairwise comparisons were made in order to identify and disentangle the different effects (Figure 4). Time effect was quantified by the differential methylation analysis performed among the control individuals (C; three different genotypes), but between the sampling times (S; three samples per genotype). This led us to perform three comparisons (C-S1 vs. C-S2, C-S2 vs. C-S3, and C-S1 vs. C-S3). Overall, time effect was associated with non-redundant (not visible in other conditions) hyper- or hypomethylations of 60 and 111 CpGs, respectively. Neither hyper- nor hypomethylation were detected in CHG or CHH contexts.

The cumulative effect of depth variation and time was quantified by differential methylation analysis performed among the individuals in the depth treatment (T; three different genotypes), but between the sampling times (S; three samples per genotype). As previously, this led us to make three pairwise comparisons (T-S1 vs. T-S2, T-S2 vs. T-S3, and T-S1 vs. T-S3). In total, 71 non-redundant hyper- and 76 non-redundant hypomethylations were identified, all in the CpG context.

The cumulative effect of depth variation and genotype was quantified by differential methylation analysis performed between treatments (depth treatment vs. control; three genotypes per condition) at the three-sampling times (T-S1 vs. C-S1, T-S2 vs. C-S2, and T-S3 vs. C-S3). These analyses revealed that: (i) in the CpG context, 2087 non-redundant cytosines were hypermethylated while 2935 non-redundant cytosines were hypomethylated; (ii) in the CHG context, 12 non-redundant cytosines were hypermethylated and 16 were hypomethylated; (iii) in the CHH context, three non-redundant cytosines were hypermethylated and three were hypomethylated.

Finally, to extract the effect of the depth variation only, we subtracted the time effect from the cumulative effect of depth and time (comparison among treatments, but between the sampling times). To do so, positions presenting differential methylation in response to the time effect were considered as non-significant when they were also differentially methylated in the cumulative effect of depth and time. None of the CpGs that were differentially methylated for the time effect were also differentially methylated for the depth variation and time effect. All the 71 hyper- and 76 hypomethylations previously identified were therefore kept for subsequent analysis.

### Enrichment Analysis and Exploration of Genes With Differentially Methylated Positions

To correlate changes in DNA methylation with changes in pigmentation, we first performed an enrichment analysis and looked for biological processes and molecular functions linked to pigmentation. As a second step, we then individually screened the genes displaying DMCpGs and searched the literature for their putative involvement in pigmentation. DMCpG that were located outside of the gene body were not considered (92 DMCpGs). Genes encoding proteins of unknown function were present in the set of genes containing DMCpGs, but the lack of functional annotation prevented us from proposing a mechanism that could explain their involvement in the phenotypic changes that occurred, although we cannot exclude that they may have a role in this phenomenon.

#### C-S1 vs. C-S2 vs. C-S3 Comparison

The GO categories significantly enriched in control conditions in relation to time were not associated with pigmentation. They were, however, associated with a seasonal effect, shown by an enrichment in GO terms linked to growth and reproduction (Supplementary Figure 2). The methylation information for all these genes and for all comparisons are provided in Supplementary Table 3.

#### T-S1 (1 Month at 8 m Depth) vs. T-S2 (1 Month at 8 m Depth Followed by 1 Month at 30 m Depth)

**FIGURE 5:**
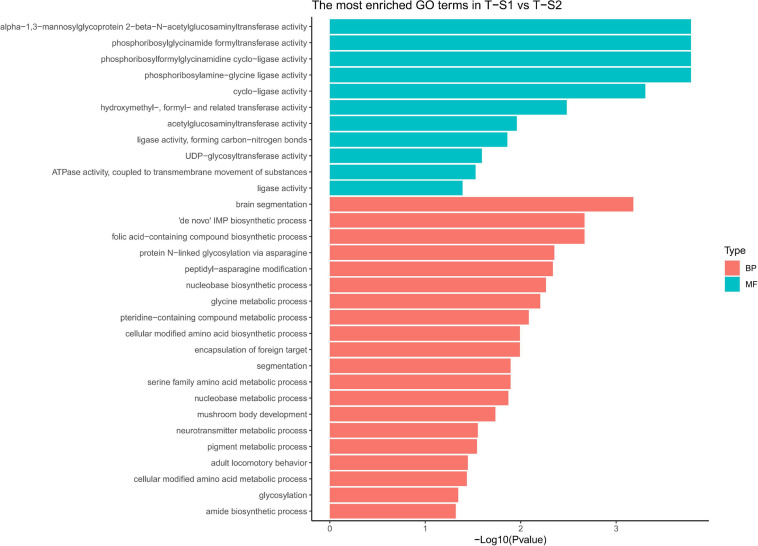
Histograms of the enriched biological processes (BP) and molecular functions (MF) from the genes with differentially methylated positions in individuals from the depth treatment samplings in T-S1 vs. T-S2 (T-S1, T-S2 = depth treatment at sampling times 1 and 2, respectively).

Gene ontology (GO) terms enrichment analysis performed for biological process category showed an enrichment of several GO terms associated with pigmentation (Figure 5): “pigment metabolic process” (GO:0042440), “pteridine-containing compound metabolic process” (GO:0042558, Frost and Malacinski, 1979), and “folic acid-containing compound biosynthetic process” (GO:0009396, Katz et al., 1987). For the molecular function category, additional GO terms linked to pigmentation processes were enriched: “UDP-glycosyltransferase activity” (GO:0008194, Vajro et al., 1995), “hydroxymethyl-formyl- and related transferase activity” (GO:0016742, Moreau et al., 2012), “alpha-1,3-mannosyl-glycoprotein 2-beta-N-acetylglucosaminyltransferase activity” (GO:0003827, Zhai et al., 2016), “phosphoribosylglycinamide formyltransferase activity” (GO:0004644), and “acetylglucosaminyltransferase activity” (GO:0016262, Chakraborty et al., 1999; Shin et al., 2015).

At the gene level (Table 2), three candidate genes were identified that had hypermethylated CpGs after the month at −30 m. The first encodes a trifunctional purine biosynthetic protein adenosine-3 (*GART*), the second an alpha-1,3-mannosyl-glycoprotein-2-beta-n-acetylglucosaminyltransferase (*MGAT1*), and the third a multidrug resistance-associated protein (*ABCC1*). *GART* is known to be involved in the synthesis of purines (purine synthesis pathway) and expression disturbances of this gene can modify the production of two pigments: melanin and pheomelanin (Amsterdam et al., 2004; Ng et al., 2009). MGAT1 is involved in the glycan synthesis pathway, and is known to be essential for shell formation in the *Pinctada* genus (Takakura et al., 2008). Finally, the *ABBC1* gene encodes an active transporter of glutathione-S-transferase (Homolya et al., 2003; Fernandes and Gattass, 2009; Rocha et al., 2014), an enzyme regulating the balance of eumelanin/pheomelanin production (Sonthalia et al., 2016).

**TABLE 2:** All significantly differentially methylated positions (*q* value < 0.05) for treatment comparisons.

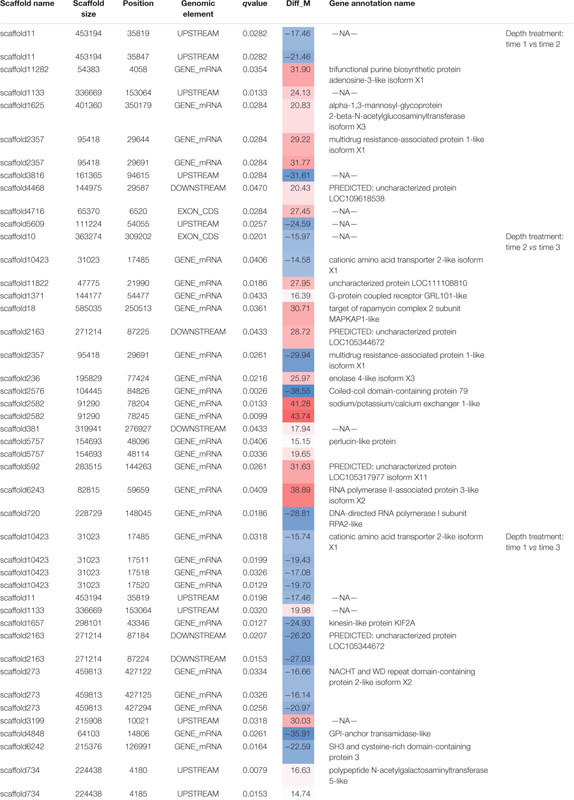

*Scaffold name: scaffold number; Scaffold size: scaffold size; Position: position of the significantly differentially methylated positions on this scaffold; Genomic element: the genomic feature containing the DNA methylation change; *q* value: *q* value; Diff-M: the differential methylation level obtained using the *Methylkit R* package; Gene annotation name: Gene annotation according to the nr and swiss-prot blast top hit. Blue shading (negative values) indicates hypomethylation of the position and red shading (positive values) hypermethylation; darker shading indicates a stronger degree of hypomethylation of hypermethylation, respectively. The last column indicates in which comparison the significantly different methylated positions were found.*

#### T-S2 (1 Month at 8 m Depth Followed by 1 Month at 30 m Depth) vs. T-S3 (1 Month at 8 m, 1 Month at 30 m, and 1 Month Back at 8 m)

**FIGURE 6:**
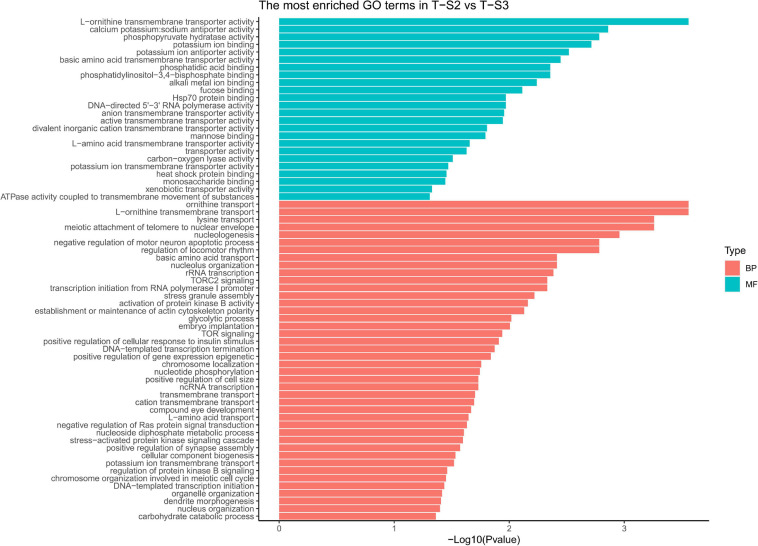
Histograms of the enriched biological processes (BP) and molecular functions (MF) from the genes with differentially methylated positions in individuals from the depth treatment samplings in T-S2 vs. T-S3 (T-S2, T-S3 = depth treatment at sampling times 2 and 3, respectively).

GO term enrichment analysis highlighted enrichment for several GO terms associated with pigmentation (Figure 6): “L-ornithine transmembrane transporter” (GO:0000064) for the biological process category; and “ornithine transport” (GO:0015822), “lysine transport” (GO:0015819), “L-amino acid transport” (GO:0015807), “basic amino acid transmembrane transporter activity” (GO:0015171), “L-amino acid transmembrane transporter activity” (GO:0015179), and “xenobiotic transporter activity” (GO:0015238) for the molecular function category.

At the gene level, 15 genes displaying DMCpGs were deemed good candidates to partly explain the phenotypic changes observed. Among the hypomethylated genes, we found those for a cationic amino acid transporter 2 (*SLC7A2*), a multidrug-associated protein (also present in the T-S1 vs. T-S2 comparison), and a coiled-coil domain-containing protein 79 (*CCDC79*). SLC7A2 is involved in the transport of arginine, lysine, and ornithine. A high number of the proteins found in the mineralized structure of *P. margaritifera* are known to be enriched in arginine and lysine, such as MSP-1, MSP-2, Aspein, Prismlain-14, and MSI60 (Addadi et al., 2006; Joubert et al., 2010; Gueguen et al., 2013). According to the NCBI GenPept database, CCDC79 can bind ion calcium, like the product of the *MGAT1* gene (see above). Among the hypermethylated genes, we found those for a G-protein coupled receptor (*GRL101*), a target of rapamycin complex 2 subunit (*MAPKAP1*), an enolase (*enolase-4*), sodium/potassium/calcium exchanger 1 and a perlucin-like protein. MAPKAP1 (TOR signaling pathway) promotes dark epithelial pigmentation (Liu et al., 2017), GRL101 (rhodopsin signaling pathway) is an ortholog of the pigment dispersing factor (Tanaka et al., 2014), and enolase (glycolysis/gluconeogenesis pathway) is a biomarker of vitiligo, a human pigmentation disorder affecting melanocytes (Hamid et al., 2015). Perlucin is a well-known matrix protein found in the nacreous layer of the pearl oyster shell (Joubert et al., 2010) with a function in the biomineralization process.

We also identified several GO terms with a less important role in pigmentation, like “TOR signaling” (GO:0031929), “TORC2 signaling” (GO:0038203), “glycolytic process” (GO:0006096), and “compound eye development” (GO:0048749) for the biological process GO category; and “calcium, potassium: sodium anti-porter activity” (GO:0005432), “alkali metal ion binding” (GO:0031420), and “mannose binding” (GO:0005537) for the molecular function GO category.

#### T-S1 (1 Month at 8 m Depth) vs. T-S3 (1 Month at 8 m, 1 Month at 30 m, and 1 Month Back at 8 m)

**FIGURE 7:**
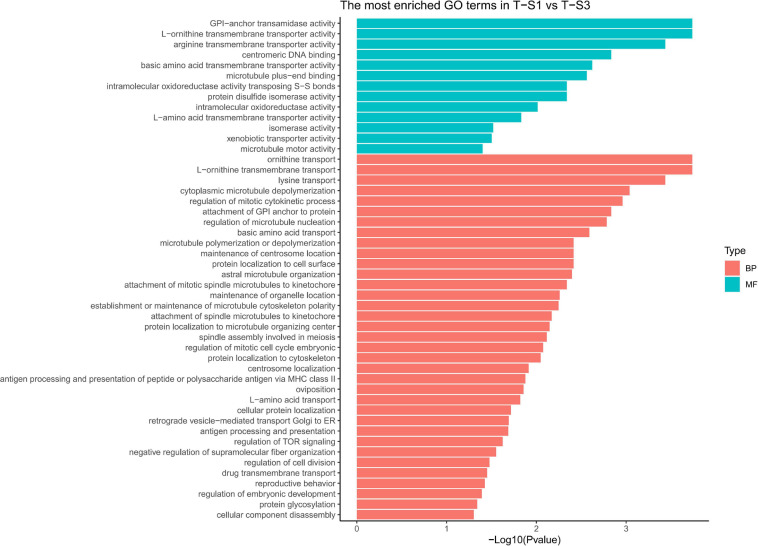
Histograms of the enriched biological processes (BP) and molecular functions (MF) from the genes with differentially methylated positions in individuals from the depth treatment samplings in T-S1 vs. T-S3 (T-S1, T-S3 = depth treatment at sampling times 1 and 3, respectively).

As the previous enrichment analyses revealed, significant enrichment of several GO categories correlated with pigmentation were identified like in the T-S2 vs. T-S3 pairwise comparison (Figure 7): “L-ornithine transmembrane transporter” (GO:0000064) for the biological process GO category; and “ornithine transport” (GO:0015822), “lysine transport” (GO:0015819), “L-amino acid transport” (GO:0015807), “basic amino acid transmembrane transporter activity” (GO:0015171), “L-amino acid transmembrane transporter activity” (GO:0015179), and “xenobiotic transporter activity” (GO:0015238) for molecular function GO category.

At the gene level, only the *GPI-anchor transamidase gene* could be linked to the pigmentation process (among other pathways) since it is involved in a human disorder characterized by altered dermal pigmentation (Ng and Freeze, 2014).

We also identified several GO terms with a less important role in pigmentation, such as the “drug transmembrane transport” (GO:0006855) for the biological process GO category; and “attachment of GPI anchor to protein” (GO:0016255), “regulation of TOR signaling” (GO:0032006), and “protein glycosylation” (GO:0006486) for the molecular function GO category.

## Discussion

Pearl farming, the second economic resource of French Polynesia, has been suffering a major economic crisis since 2001. To address this problem, stakeholders, together with pearl farmers and scientists, are developing an ambitious plan to reduce the volume of pearl production, but to increase pearl quality. This objective could be reached through one of the most economically interesting traits of *Pinctada margaritifera*: its ability to express the largest range of inner shell color (and thus pearl color) of any pearl-producing species worldwide (Ky et al., 2014; Stenger et al., 2019). Indeed, producing unique, highly valuable, pearls displaying a palette of phenotypes ranging from very dark to very pale colors constitutes an efficient way to diversify the production and appeal to different markets. Selection of donor oysters based on color phenotype was started a few years ago (Ky et al., 2013). However, the possibility of acting directly on the selected oysters to enhance their color and quality as donors is also an attractive method for improving pearl quality. For this, a better understanding of the interactions between the phenotype and gene expression correlated with environmental conditions is essential (Gavery and Roberts, 2017), and would allow the development of epigenetic marker-assisted selection or, more generally, epigenetics-assisted cultural practices.

As a first step to understanding the mechanisms behind this environmentally induced color variation, we applied an environmental forcing (culture-depth variation) known to affect pearl and inner-shell darkness (Stenger et al., 2019), and studied, under constant genotype, the DNA methylation changes induced. In addition to providing the first description of a pearl oyster methylome, our analyses identified specific methylation changes that affected candidate genes involved in the expression of shell and pearl color darkness. These genes were involved in both pigmentation (biological coloring mechanisms) and iridescence (physical coloring mechanisms based on the differential organization of biomineral crystals).

### The First Methylome of the *Pterioidea* Super-Family Shows Similar Characteristics to Other Invertebrate Methylomes

The present study provides to our knowledge the first methylome of a member of the *Pterioidea* super-family. The whole-genome bisulfite sequencing (WGBS-seq) of the pearl oyster mantle tissues revealed that its methylome is of the mosaic type and similar in many ways to what is classically described in some other invertebrates. Indeed, *P. margaritifera* mainly displays cytosine methylation in the CpG context, as already described in *Crassostrea gigas* (Thunberg, 1793) by Gavery and Roberts (2013) and in *Biomphalaria glabrata* (Say, 1818) by Adema et al. (2017). Likewise, differential methylation was also mainly identified in the CpG context, as in Wang et al. (2014) who reported that more than 99% of DNA methylation changes were restricted to the CpG context in *C. gigas*. The pattern of methylation we obtained occurred essentially in the gene bodies, with some accretion upstream and downstream of the gene, as described for mosaic methylation in other mollusks (Sarda et al., 2012; Wang et al., 2014; Rondon et al., 2017). These features of a short, but densely methylated region (corresponding to the genes), interspersed by long unmethylated regions (intergenic) are characteristic of the mosaic pattern of DNA methylation (Gavery and Roberts, 2013), which shows the typical bimodal distribution generally met in invertebrates. The lowly methylated genes were involved in the reproductive process, cellular signaling, and environmentally responsive functions. The low methylation of genes involved in the response to environmental changes is something reported in many different invertebrates (Sarda et al., 2012), but the presence of functions associated with reproduction is less common. It may be explained by the tissue used to produce this methylome, the mantle, which is a tissue not involved in reproduction (see correlation between methylation levels and gene expression Figure 2D). The genes identified in highly methylated regions were essentially involved in housekeeping functions (Gavery and Roberts, 2013; Olson and Roberts, 2014; Wang et al., 2014). When comparing the gene body methylation rate with the gene expression level, we demonstrated that moderately methylated genes have higher expression levels than lowly or highly methylated ones. This result is consistent with other studies (Feng et al., 2010; Xiang et al., 2010; Zemach et al., 2010) and strengthens the emerging hypothesis that, in invertebrates, gene expression and gene body methylation functions as a negative feedback loop in which gene expression increases with gene methylation until reaching a tipping point where additional methylation decreases transcription (Dixon et al., 2018).

### Environmentally Induced DNA Methylation Changes and Their Link With Pigmentation

Among the DNA methylation changes that occurred during our experiment, several occurred in genes known to be involved in pigmentation pathways. Among these, the pteridine pathway was recently identified as a key player in the expression of the yellow color phenotype in *P. margaritifera* (Stenger et al., in press). Indeed, different derivates of pteridine can lead to the production of sepiapterin and xanthopterins, two yellow pigments (Ng et al., 2009). The folic acid pathway is also significantly affected by DNA methylation changes. Although its involvement in molluscan pigmentation is unknown, folic acid deficiency is linked to melanosis (e.g., melanin overproduction) in mammals, which results in a black pigmentation (Sharp et al., 1980). Since the implication of melanin in pearl oyster pigmentation has previously been identified (Lemer et al., 2015), an epigenetically driven modification of gene expression in the folic-acid pathway could be associated with the darkening color phenotypes expressed in response to an increase in depth.

**FIGURE 8:**
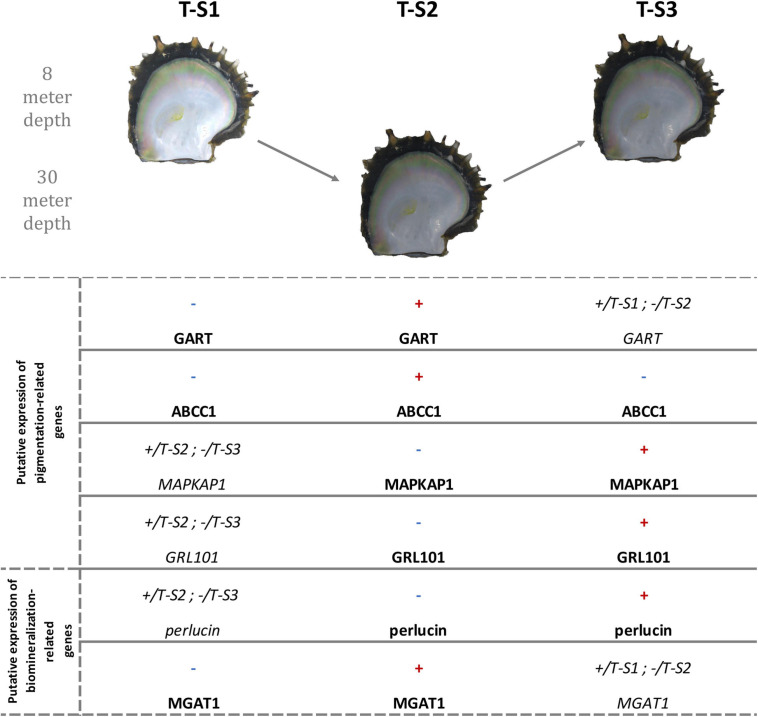
Methylation direction changes in candidate genes linked to inner shell color darkening in the depth treatment individuals at the three successive sampling times (T-S1, T-S2, and T-S3). The first four genes are involved in pigmentation processes, and the last two in biomineralization processes. Red “+” illustrates a significant hypermethylation of at least one CpG and blue “-” illustrates a significant hypomethylation of at least one CpG. +/T-S(a) and -/T-S(b) mean that the methylation in the present sampling time is more methylated than in the T-S(a) and less than in the T-S(b). *GART*, trifunctional purine biosynthetic protein adenosine-3; *ABCC1*, multidrug resistance-associated protein 1; *MAPKAP1*, target of rapamycin complex 2 subunit *MAPKAP1*; *GRL101*, G-protein coupled receptor *GRL101*; *MGAT1*, alpha-1,3-mannosyl-glycoprotein-2-beta-n-acetylglucosaminyltransferase. Pictures showed are illustrative.

At the gene level, *GART*, a hypermethylated gene included in three enriched GO categories (Figure 8), encodes a trifunctional purine biosynthetic protein, adenosine-3. This protein is involved in the *de novo* purine synthesis pathway (Amsterdam et al., 2004) and is composed of three subunits (a phosphoribosylglycinamide formyltransferase, a phosphoribosylglycinamide synthetase, and a phosphoribosylaminoimidazole synthetase). Biochemically, it catalyzes steps 2, 3, and 5 of inosine monophosphate (IMP) synthesis (Amsterdam et al., 2004; Ng et al., 2009). IMP is one of the precursors initiating the pterin and the Raper-Manson pathways, two pathways leading to pigmentation in *P. margaritifera* (Stenger et al., in press). Ng et al. (2009) have shown that mutations in *GART* are associated with pigmentation defects in juvenile zebrafish *Danio rerio* (Buchanan-Hamilton, 1822) due to disturbances of the pterin and Rapper-Mason pathways. Wild-type zebrafish are mainly yellow with black spots, while Δ-GART juveniles are entirely black (Ng et al., 2009). We can, therefore, hypothesize that methylation changes in the *GART* gene may affect its expression, subsequently affecting the pterin and Rapper-Mason pathways and leading to a darkening of the shell.

The gene for multidrug resistance-associated protein 1 (*ABCC1*) presented two hypermethylated positions after the period at 30 m, a methylation state that reverted after the return to 8 m. *ABCC1* is known to mediate ATP-dependent transport of glutathione and glutathione conjugates (Homolya et al., 2003). In a previous study it was proposed that glutathione plays an essential role in the expression of the yellow and black pigments (Stenger et al., in press). Glutathione-S-transferase (GST) activity is central in regulating the production of the yellow pheomelanin and black eumelanin pigments through the Raper-Manson pathway (Sonthalia et al., 2016). Methylation changes in the *ABCC1* gene could therefore promote variation in the quantity of glutathione available and modify the regulation of the production of pheomelanin and eumelanin. An overproduction of eumelanin may explain the observed darkening of the shell.

*GRL101* presented a hypermethylated response to the return to 8 m. According to Tanaka et al. (2014), this gene is an ortholog of the pigment dispersing factor, a gene responsible for changes in the concentration of chromatophoral pigment in response to darkness (Rao and Riehm, 1993). In crustaceans, it was proposed that color variation due to changing light conditions was caused by the dispersion of retinal chromatophore pigments linked with the activation of *GRL101* (Rao and Riehm, 1993; Auerswald et al., 2008). The methylation change of *GRL101*, the similarities between the environmental triggers (a decrease of light) activating *GRL101* in other organisms, and the phenotypes resulting from this activation argue in favor of the involvement of *GRL101* in *P. margaritifera* color variation.

The *GPI-anchor transamidase-like* gene was hypomethylated after the return to 8 m depth. According to Ng and Freeze (2014), a mutation of the *GPI-anchor transamidase* genes is involved in a human disorder characterized by an altered dermal pigmentation (Ng and Freeze, 2014). This was later confirmed by RNAi experiments targeting GPI-anchor transamidase transcripts and resulting in a hyper-pigmented dark swellings in the maize anthracnose fungus *Colletotrichum graminicola* (G.W. Wilson 1914) (Oliveira-Garcia and Deising, 2016).

*Enolase-4* is another candidate gene displaying hypomethylation at 30 m. Enolases are metalloenzymes involved in glycolysis and glycogen storage. One study reported a correlation between enolase activity and pigmentation: Hamid et al. (2015) showed that patients with vitiligo (a pigmentation disorder affecting melanocytes and inhibiting pigment synthesis) synthesize antibodies directed against enolases. Since their discovery, enolases have been used as biomarkers for the diagnosis, treatment, and monitoring of vitiligo (Hamid et al., 2015). The causes of this pathology are still unclear, although both genetic and environmental factors seem to be involved (Hamid et al., 2015). In the case of the pearl oyster, the hypomethylation of the enolase gene at 30 m may be associated with a change of expression inducing a darker phenotype.

The last of the genes subject to methylation change (hypermethylation after the last period at 8 m) and displaying a functional link with pigmentation is the target of rapamycin complex 2 subunit (*MAPKAP1*). This gene is involved in the TORC2 and TOR signaling pathways. The activation of these signaling pathways is known to promote a dark epithelial pigmentation due to the proliferation and the migration of retinal pigmentation epithelial cells (RPE cells) (Liu et al., 2017).

Among the six genes displaying a functional link with the pigmentation process or its regulation, four (*GART*, *ABCC1*, *MAPKAP1*, and *GRL101*) were associated with the expression of darker phenotypes, while the two others were associated with pigmentation disorders. Further experiments will be necessary to confirm and define their role, such as gene expression quantification, RNAi, and, once possible, genome editing, and epigenetic engineering. Thus, our results provide the first step toward this new research field.

### Biomineralization and Pearl Darkness

In addition to a darkening of the coloration (Stenger et al., 2019), previous experiments have shown that a variation in depth also affects the shape and size of the aragonite tablets of the shell of *P. margaritifera* (Rousseau and Rollion-Bard, 2012). Aragonite tablets are the structural unit of nacre, the CaCO_3_ polymorph that constitutes the inner-shell of pearl oyster and the pearl itself (Rousseau and Rollion-Bard, 2012). Variation in the organization of these aragonite tablets can induce a change in color and luster (brightness) due to a change in the physical iridescence (Liu et al., 1999; Wang et al., 2008). Although not yet demonstrated, it is suspected that pigments contributing to nacre color are constituents of the intra-lamellar silk-fibroin gel that is localized between aragonite tablets (Addadi et al., 2006). Variation in the size of these tablets could therefore lead to a variation in the quantity of pigments that can be viewed through the last biomineralized aragonite layers. Interestingly, among the genes displaying methylation changes in response to a variation in water depth, two are well-known actors of the biomineralization processes of the nacreous layer: *perlucin* (Joubert et al., 2010) and *MGAT1* (Takakura et al., 2008).

Perlucin is a protein found in the shell organic matrix of several Mollusca, including *P. margaritifera* (Weiss et al., 2000; Blank et al., 2003; Marie et al., 2013; Joubert et al., 2014). Experiments with purified Mollusca perlucin have suggested its involvement in calcium carbonate precipitation by favoring nucleation, crystallization and crystal growth control (Weiss et al., 2000). A variation in its expression can therefore have a huge effect on aragonite tablet size and organization (Rousseau and Rollion-Bard, 2012) and may thus modify the iridescence and transparency properties of the top aragonite layers (Rousseau and Rollion-Bard, 2012).

*MGAT1* is a gene whose product initiates carbohydrate formation and is essential for the conversion of high-mannose to hybrid and complex N-glycans. This protein is involved in the protein glycosylation pathway, which is part of Protein modification. Interestingly, Takakura et al. (2008) identified an acidic N-glycan post-translationally attached to nacrein in *Pinctada fucata* that allows calcium binding. Moreover, nacrein is one of the main proteins found in the nacreous part of the shell (Rousseau and Rollion-Bard, 2012). So, although the *MGAT1* gene plays no role in crystal formation, a possible link affecting nacrein formation can still be found. Future proteomics studies will be necessary to better uncover the role of *MGAT1* in *P. margaritifera* shell coloration.

### Epigenetics and Pearl Culture

As previously reported, the yo-yo experiment resulted in a general darkening of the inner shell of *P. margaritifera* in response to an increase in depth (Stenger et al., 2019), an environmentally induced phenotype that was maintained even after a return to the control depth (8 m). Such an enduring phenotypic response could be considered as good evidence of the involvement of epigenetic control (Sutherland and Costa, 2003; Harris et al., 2012; Bräutigam et al., 2013). The maintenance of this phenotype was previously documented in this species cultured for pearl production, and can last for over 18 months (Stenger et al., 2019). In another biological model, maize, stress-induced hypermethylation of *P-pr* (Richards, 2006; Lukens and Zhan, 2007) was associated with a reduced pigmentation that lasted in some cases for the entire life of an individual, and could even be transmitted to the next generation (Richards, 2006; Bossdorf et al., 2008). Such effects could offer huge benefits for pearl farming. First, this long-lasting effect suggests that farmers could better control their production through dedicated conditioning of recipient and/or donor oysters. Additionally, the transgenerational effect described for maize, although not yet tested for pearl oysters, suggests that epigenetic marker-assisted selection could be envisioned. Such an approach may offer the possibility of selecting phenotypes of interest without the associated risk of eroding genetic diversity and/or the integration into natural populations of spat produced by farmed oysters (Reisser et al., 2020).

## Data Availability Statement

The datasets generated for this study can be found on the NCBI (BioProject PRJNA663978). Scripts used are provided on GitHub (PLStenger/Pearl_Oyster_Colour_BS_Seq/00_scripts).

## Author Contributions

JV-D, P-LS, SP, and C-LK designed the study. P-LS and MM performed the experiments. P-LS, CC, and CG analyzed the data. P-LS and JV-D drafted the manuscript. Funding was obtained by CL-K, SP, and JV-D. All authors approved the manuscript.

## Conflict of Interest

The authors declare that the research was conducted in the absence of any commercial or financial relationships that could be construed as a potential conflict of interest.

## Footnotes

1 https://wwz.ifremer.fr/pcdm/Equipement

## Supplementary Material

Supplementary Figure 1 Treemaps of biological processes obtained from the GO terms significantly enriched from the lowly methylated genes **(A)** (data from Figure 2C – “1” part) and the highly methylated genes **(B)** (data from Figure 2C – “2” part).

Supplementary Figure 2 Treemaps of biological processes and molecular functions obtained from the GO terms significantly enriched from the genes with differentially methylated positions in control individuals (C-S1, control in time 1; C-S2, control in time 2; C-S3, control in time 3).

Supplementary Table 1 FastQC results for raw and trimmed data.

Supplementary Table 2 Average values by category (control/depth treatment) and sampling time for: the number of read pairs, paired-end alignments, mapping efficiency, number of duplicated alignments removed after mapping, the number of C in sequences, the number of C in percentage in CpG, CHG, CHH or in unknown contexts, and the basic statistics of the gene body methylation rate (GBM – on the 54408 genes of the reference genome).

Supplementary Table 3 All significantly differentially methylated positions (*q* value < 0.05) for the controls. Scaffold name: scaffold number; Scaffold size: scaffold size; Position: position of the significantly differentially methylated positions on this scaffold; Genomic element: the genomic feature containing the DNA methylation change; *q* value: *q* value; Diff-M: the differential methylation level obtained using the *Methylkit R* package; Gene annotation name: Gene annotation according to the nr and swiss-prot blast top hit. Blue shading (negative values) indicates hypomethylation of the position and red shading (positive values) hypermethylation; darker shading indicates a stronger degree of hypomethylation of hypermethylation, respectively. The last column indicates in which comparison the significantly different methylated positions were found.
