# Supplementary figures and images for: Environmentally Driven Color Variation in the Pearl Oyster Pinctada margaritifera var. cumingii (Linnaeus, 1758) Is Associated With Differential Methylation of CpGs in Pigment- and Biomineralization-Related Genes (part 1 of 2)
Source: Front Genet. 2021 Mar 19;12:630290. doi: 10.3389/fgene.2021.630290 (PMC8018223; doi:10.3389/fgene.2021.630290)

## Slide 1
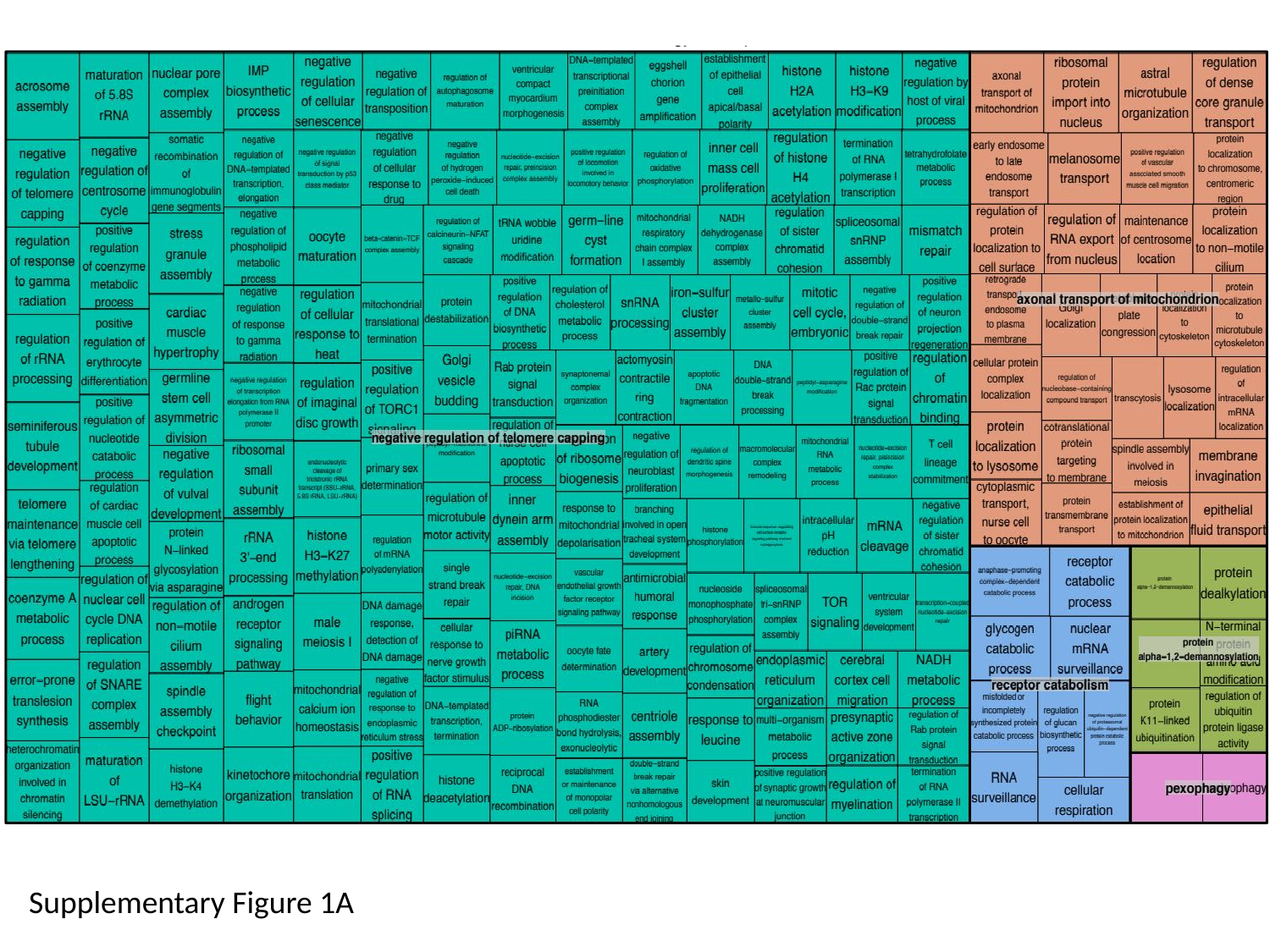

Supplementary Figure 1A

## Slide 2
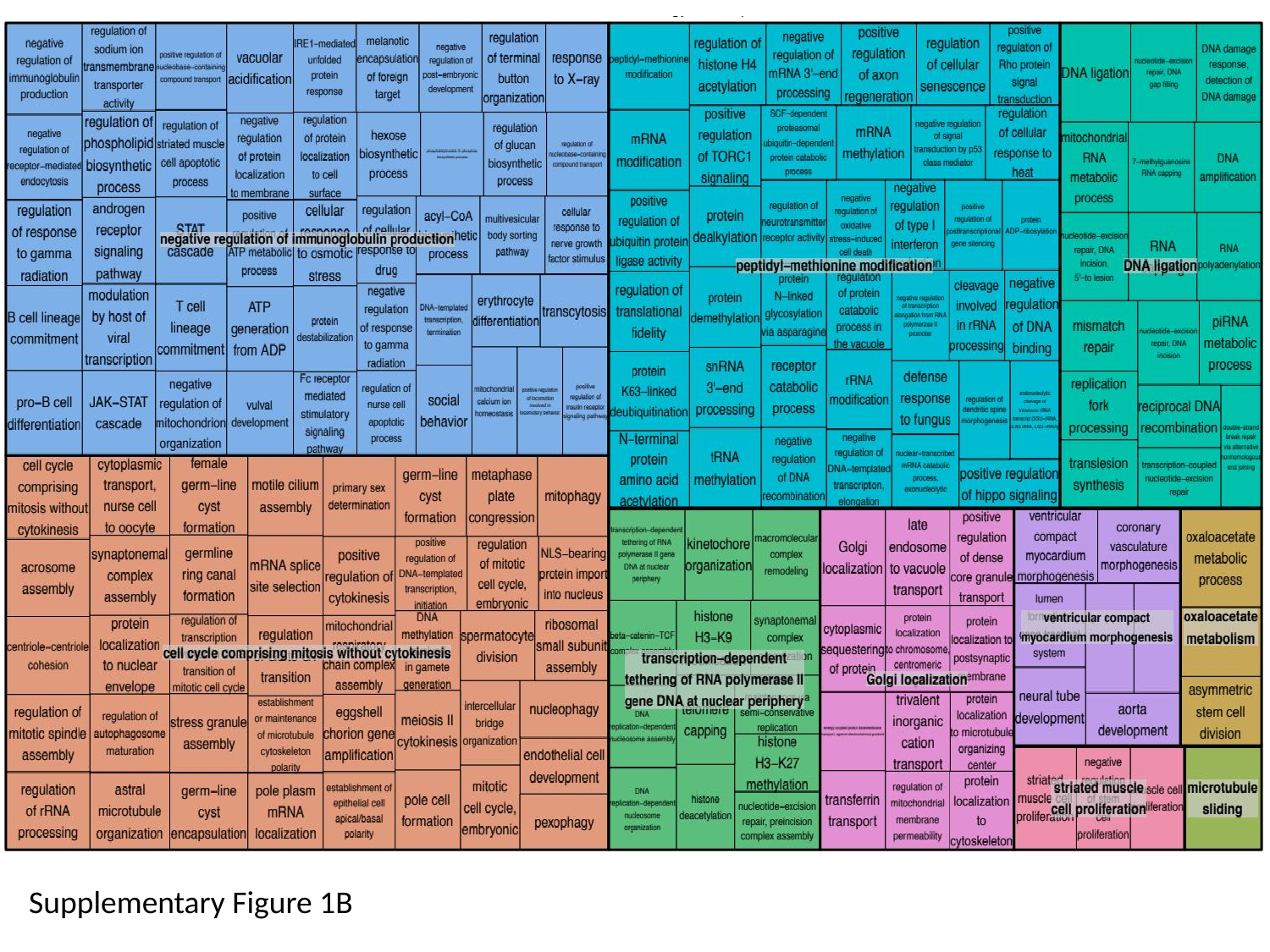

Supplementary Figure 1B

## Slide 3
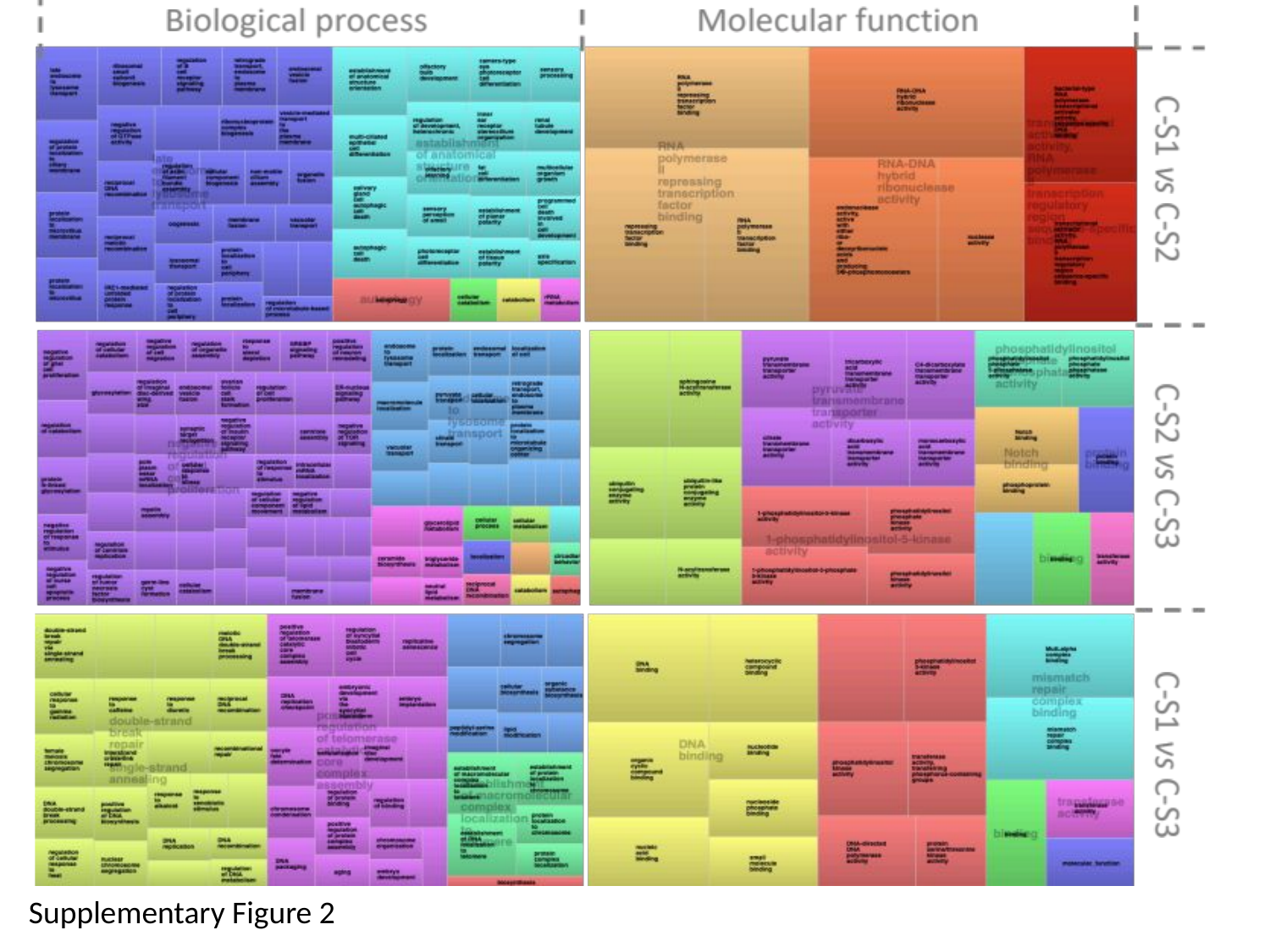

Supplementary Figure 2

Supplement: Supplementary Figure 1 — Treemaps of biological processes obtained from the GO terms significantly enriched from the lowly methylated genes (A) (data from Figure 2C – “1” part) and the highly methylated genes (B) (data from Figure 2C – “2” part). [file Presentation_1.PPTX]

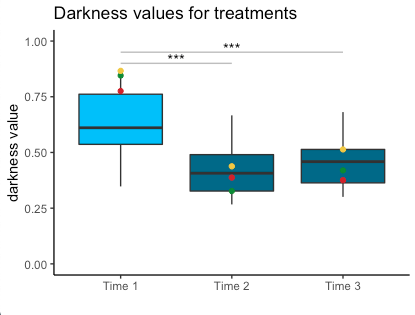

Supplement: Supplementary file 5 [file Data_Sheet_1.ZIP › ImaginR_raw_data_screenshots/Capture dΓÇÖe╠ücran 2019-08-08 a╠Ç 12.21.58.png]

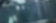

Supplement: Supplementary file 5 [file Data_Sheet_1.ZIP › ImaginR_raw_data_screenshots/3-Videos_8_m-2017-08-17_3eme_echantillonnage/Screenshots/620/620-9.jpg]

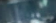

Supplement: Supplementary file 5 [file Data_Sheet_1.ZIP › ImaginR_raw_data_screenshots/3-Videos_8_m-2017-08-17_3eme_echantillonnage/Screenshots/620/620-8.jpg]

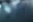

Supplement: Supplementary file 5 [file Data_Sheet_1.ZIP › ImaginR_raw_data_screenshots/3-Videos_8_m-2017-08-17_3eme_echantillonnage/Screenshots/620/620-6.png]

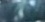

Supplement: Supplementary file 5 [file Data_Sheet_1.ZIP › ImaginR_raw_data_screenshots/3-Videos_8_m-2017-08-17_3eme_echantillonnage/Screenshots/620/620-7.jpg]

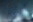

Supplement: Supplementary file 5 [file Data_Sheet_1.ZIP › ImaginR_raw_data_screenshots/3-Videos_8_m-2017-08-17_3eme_echantillonnage/Screenshots/620/620-5.jpg]

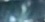

Supplement: Supplementary file 5 [file Data_Sheet_1.ZIP › ImaginR_raw_data_screenshots/3-Videos_8_m-2017-08-17_3eme_echantillonnage/Screenshots/620/620-4.jpg]

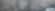

Supplement: Supplementary file 5 [file Data_Sheet_1.ZIP › ImaginR_raw_data_screenshots/3-Videos_8_m-2017-08-17_3eme_echantillonnage/Screenshots/007/007-10.jpg]

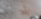

Supplement: Supplementary file 5 [file Data_Sheet_1.ZIP › ImaginR_raw_data_screenshots/3-Videos_8_m-2017-08-17_3eme_echantillonnage/Screenshots/007/007-1.jpg]

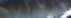

Supplement: Supplementary file 5 [file Data_Sheet_1.ZIP › ImaginR_raw_data_screenshots/3-Videos_8_m-2017-08-17_3eme_echantillonnage/Screenshots/007/007-2.jpg]

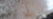

Supplement: Supplementary file 5 [file Data_Sheet_1.ZIP › ImaginR_raw_data_screenshots/3-Videos_8_m-2017-08-17_3eme_echantillonnage/Screenshots/007/007-3.jpg]

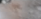

Supplement: Supplementary file 5 [file Data_Sheet_1.ZIP › ImaginR_raw_data_screenshots/3-Videos_8_m-2017-08-17_3eme_echantillonnage/Screenshots/007/007-7.jpg]

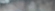

Supplement: Supplementary file 5 [file Data_Sheet_1.ZIP › ImaginR_raw_data_screenshots/3-Videos_8_m-2017-08-17_3eme_echantillonnage/Screenshots/007/007-6.jpg]

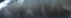

Supplement: Supplementary file 5 [file Data_Sheet_1.ZIP › ImaginR_raw_data_screenshots/3-Videos_8_m-2017-08-17_3eme_echantillonnage/Screenshots/007/007-8.jpg]

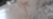

Supplement: Supplementary file 5 [file Data_Sheet_1.ZIP › ImaginR_raw_data_screenshots/3-Videos_8_m-2017-08-17_3eme_echantillonnage/Screenshots/007/007-9.jpg]

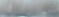

Supplement: Supplementary file 5 [file Data_Sheet_1.ZIP › ImaginR_raw_data_screenshots/3-Videos_8_m-2017-08-17_3eme_echantillonnage/Screenshots/180/180-6.jpg]

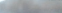

Supplement: Supplementary file 5 [file Data_Sheet_1.ZIP › ImaginR_raw_data_screenshots/3-Videos_8_m-2017-08-17_3eme_echantillonnage/Screenshots/180/180-7.jpg]

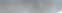

Supplement: Supplementary file 5 [file Data_Sheet_1.ZIP › ImaginR_raw_data_screenshots/3-Videos_8_m-2017-08-17_3eme_echantillonnage/Screenshots/180/180-5.jpg]

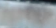

Supplement: Supplementary file 5 [file Data_Sheet_1.ZIP › ImaginR_raw_data_screenshots/3-Videos_8_m-2017-08-17_3eme_echantillonnage/Screenshots/180/180-4.jpg]

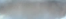

Supplement: Supplementary file 5 [file Data_Sheet_1.ZIP › ImaginR_raw_data_screenshots/3-Videos_8_m-2017-08-17_3eme_echantillonnage/Screenshots/180/180-1.jpg]

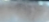

Supplement: Supplementary file 5 [file Data_Sheet_1.ZIP › ImaginR_raw_data_screenshots/3-Videos_8_m-2017-08-17_3eme_echantillonnage/Screenshots/180/180-3.jpg]

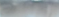

Supplement: Supplementary file 5 [file Data_Sheet_1.ZIP › ImaginR_raw_data_screenshots/3-Videos_8_m-2017-08-17_3eme_echantillonnage/Screenshots/180/180-2.jpg]

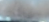

Supplement: Supplementary file 5 [file Data_Sheet_1.ZIP › ImaginR_raw_data_screenshots/3-Videos_8_m-2017-08-17_3eme_echantillonnage/Screenshots/180/180-9.jpg]

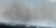

Supplement: Supplementary file 5 [file Data_Sheet_1.ZIP › ImaginR_raw_data_screenshots/3-Videos_8_m-2017-08-17_3eme_echantillonnage/Screenshots/180/180-8.jpg]

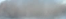

Supplement: Supplementary file 5 [file Data_Sheet_1.ZIP › ImaginR_raw_data_screenshots/3-Videos_8_m-2017-08-17_3eme_echantillonnage/Screenshots/180/180-10.jpg]

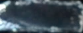

Supplement: Supplementary file 5 [file Data_Sheet_1.ZIP › ImaginR_raw_data_screenshots/3-Videos_8_m-2017-08-17_3eme_echantillonnage/Screenshots/613/613-10.jpg]

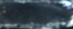

Supplement: Supplementary file 5 [file Data_Sheet_1.ZIP › ImaginR_raw_data_screenshots/3-Videos_8_m-2017-08-17_3eme_echantillonnage/Screenshots/613/613-1.jpg]

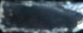

Supplement: Supplementary file 5 [file Data_Sheet_1.ZIP › ImaginR_raw_data_screenshots/3-Videos_8_m-2017-08-17_3eme_echantillonnage/Screenshots/613/613-3.jpg]

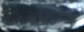

Supplement: Supplementary file 5 [file Data_Sheet_1.ZIP › ImaginR_raw_data_screenshots/3-Videos_8_m-2017-08-17_3eme_echantillonnage/Screenshots/613/613-2.jpg]

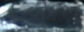

Supplement: Supplementary file 5 [file Data_Sheet_1.ZIP › ImaginR_raw_data_screenshots/3-Videos_8_m-2017-08-17_3eme_echantillonnage/Screenshots/613/613-5.jpg]

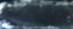

Supplement: Supplementary file 5 [file Data_Sheet_1.ZIP › ImaginR_raw_data_screenshots/3-Videos_8_m-2017-08-17_3eme_echantillonnage/Screenshots/613/613-9.jpg]

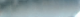

Supplement: Supplementary file 5 [file Data_Sheet_1.ZIP › ImaginR_raw_data_screenshots/3-Videos_8_m-2017-08-17_3eme_echantillonnage/Screenshots/002/002-6.jpg]

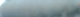

Supplement: Supplementary file 5 [file Data_Sheet_1.ZIP › ImaginR_raw_data_screenshots/3-Videos_8_m-2017-08-17_3eme_echantillonnage/Screenshots/002/002-7.jpg]

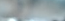

Supplement: Supplementary file 5 [file Data_Sheet_1.ZIP › ImaginR_raw_data_screenshots/3-Videos_8_m-2017-08-17_3eme_echantillonnage/Screenshots/002/002-5.jpg]

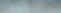

Supplement: Supplementary file 5 [file Data_Sheet_1.ZIP › ImaginR_raw_data_screenshots/3-Videos_8_m-2017-08-17_3eme_echantillonnage/Screenshots/002/002-1.jpg]

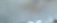

Supplement: Supplementary file 5 [file Data_Sheet_1.ZIP › ImaginR_raw_data_screenshots/3-Videos_8_m-2017-08-17_3eme_echantillonnage/Screenshots/002/002-3.jpg]

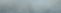

Supplement: Supplementary file 5 [file Data_Sheet_1.ZIP › ImaginR_raw_data_screenshots/3-Videos_8_m-2017-08-17_3eme_echantillonnage/Screenshots/002/002-10.jpg]

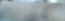

Supplement: Supplementary file 5 [file Data_Sheet_1.ZIP › ImaginR_raw_data_screenshots/3-Videos_8_m-2017-08-17_3eme_echantillonnage/Screenshots/002/002-9.jpg]

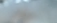

Supplement: Supplementary file 5 [file Data_Sheet_1.ZIP › ImaginR_raw_data_screenshots/3-Videos_8_m-2017-08-17_3eme_echantillonnage/Screenshots/002/002-8.jpg]

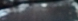

Supplement: Supplementary file 5 [file Data_Sheet_1.ZIP › ImaginR_raw_data_screenshots/3-Videos_8_m-2017-08-17_3eme_echantillonnage/Screenshots/183/183-7.jpg]

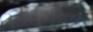

Supplement: Supplementary file 5 [file Data_Sheet_1.ZIP › ImaginR_raw_data_screenshots/3-Videos_8_m-2017-08-17_3eme_echantillonnage/Screenshots/183/183-6.jpg]

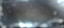

Supplement: Supplementary file 5 [file Data_Sheet_1.ZIP › ImaginR_raw_data_screenshots/3-Videos_8_m-2017-08-17_3eme_echantillonnage/Screenshots/183/183-4.jpg]

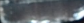

Supplement: Supplementary file 5 [file Data_Sheet_1.ZIP › ImaginR_raw_data_screenshots/3-Videos_8_m-2017-08-17_3eme_echantillonnage/Screenshots/183/183-5.jpg]

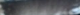

Supplement: Supplementary file 5 [file Data_Sheet_1.ZIP › ImaginR_raw_data_screenshots/3-Videos_8_m-2017-08-17_3eme_echantillonnage/Screenshots/183/183-1.jpg]

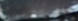

Supplement: Supplementary file 5 [file Data_Sheet_1.ZIP › ImaginR_raw_data_screenshots/3-Videos_8_m-2017-08-17_3eme_echantillonnage/Screenshots/183/183-2.jpg]

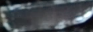

Supplement: Supplementary file 5 [file Data_Sheet_1.ZIP › ImaginR_raw_data_screenshots/3-Videos_8_m-2017-08-17_3eme_echantillonnage/Screenshots/183/183-3.jpg]

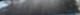

Supplement: Supplementary file 5 [file Data_Sheet_1.ZIP › ImaginR_raw_data_screenshots/3-Videos_8_m-2017-08-17_3eme_echantillonnage/Screenshots/183/183-10.jpg]

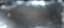

Supplement: Supplementary file 5 [file Data_Sheet_1.ZIP › ImaginR_raw_data_screenshots/3-Videos_8_m-2017-08-17_3eme_echantillonnage/Screenshots/183/183-8.jpg]

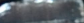

Supplement: Supplementary file 5 [file Data_Sheet_1.ZIP › ImaginR_raw_data_screenshots/3-Videos_8_m-2017-08-17_3eme_echantillonnage/Screenshots/183/183-9.jpg]

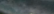

Supplement: Supplementary file 5 [file Data_Sheet_1.ZIP › ImaginR_raw_data_screenshots/2-Videos_30_m/Screenshots/620/620-9.jpg]

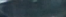

Supplement: Supplementary file 5 [file Data_Sheet_1.ZIP › ImaginR_raw_data_screenshots/2-Videos_30_m/Screenshots/620/620-8.jpg]

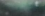

Supplement: Supplementary file 5 [file Data_Sheet_1.ZIP › ImaginR_raw_data_screenshots/2-Videos_30_m/Screenshots/620/620-6.jpg]

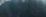

Supplement: Supplementary file 5 [file Data_Sheet_1.ZIP › ImaginR_raw_data_screenshots/2-Videos_30_m/Screenshots/620/620-7.jpg]

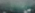

Supplement: Supplementary file 5 [file Data_Sheet_1.ZIP › ImaginR_raw_data_screenshots/2-Videos_30_m/Screenshots/620/620-5.jpg]

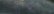

Supplement: Supplementary file 5 [file Data_Sheet_1.ZIP › ImaginR_raw_data_screenshots/2-Videos_30_m/Screenshots/620/620-4.jpg]

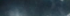

Supplement: Supplementary file 5 [file Data_Sheet_1.ZIP › ImaginR_raw_data_screenshots/2-Videos_30_m/Screenshots/620/620-1.jpg]

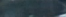

Supplement: Supplementary file 5 [file Data_Sheet_1.ZIP › ImaginR_raw_data_screenshots/2-Videos_30_m/Screenshots/620/620-3.jpg]

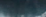

Supplement: Supplementary file 5 [file Data_Sheet_1.ZIP › ImaginR_raw_data_screenshots/2-Videos_30_m/Screenshots/620/620-2.jpg]

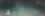

Supplement: Supplementary file 5 [file Data_Sheet_1.ZIP › ImaginR_raw_data_screenshots/2-Videos_30_m/Screenshots/620/620-10.jpg]

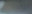

Supplement: Supplementary file 5 [file Data_Sheet_1.ZIP › ImaginR_raw_data_screenshots/2-Videos_30_m/Screenshots/007/007-10.jpg]

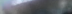

Supplement: Supplementary file 5 [file Data_Sheet_1.ZIP › ImaginR_raw_data_screenshots/2-Videos_30_m/Screenshots/007/007-1.jpg]

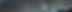

Supplement: Supplementary file 5 [file Data_Sheet_1.ZIP › ImaginR_raw_data_screenshots/2-Videos_30_m/Screenshots/007/007-2.jpg]

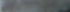

Supplement: Supplementary file 5 [file Data_Sheet_1.ZIP › ImaginR_raw_data_screenshots/2-Videos_30_m/Screenshots/007/007-3.jpg]

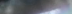

Supplement: Supplementary file 5 [file Data_Sheet_1.ZIP › ImaginR_raw_data_screenshots/2-Videos_30_m/Screenshots/007/007-7.jpg]

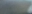

Supplement: Supplementary file 5 [file Data_Sheet_1.ZIP › ImaginR_raw_data_screenshots/2-Videos_30_m/Screenshots/007/007-6.jpg]

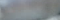

Supplement: Supplementary file 5 [file Data_Sheet_1.ZIP › ImaginR_raw_data_screenshots/2-Videos_30_m/Screenshots/007/007-4.jpg]

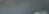

Supplement: Supplementary file 5 [file Data_Sheet_1.ZIP › ImaginR_raw_data_screenshots/2-Videos_30_m/Screenshots/007/007-5.jpg]

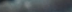

Supplement: Supplementary file 5 [file Data_Sheet_1.ZIP › ImaginR_raw_data_screenshots/2-Videos_30_m/Screenshots/007/007-8.jpg]

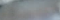

Supplement: Supplementary file 5 [file Data_Sheet_1.ZIP › ImaginR_raw_data_screenshots/2-Videos_30_m/Screenshots/007/007-9.jpg]

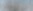

Supplement: Supplementary file 5 [file Data_Sheet_1.ZIP › ImaginR_raw_data_screenshots/2-Videos_30_m/Screenshots/180/180-6.jpg]

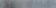

Supplement: Supplementary file 5 [file Data_Sheet_1.ZIP › ImaginR_raw_data_screenshots/2-Videos_30_m/Screenshots/180/180-7.jpg]

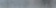

Supplement: Supplementary file 5 [file Data_Sheet_1.ZIP › ImaginR_raw_data_screenshots/2-Videos_30_m/Screenshots/180/180-5.jpg]

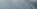

Supplement: Supplementary file 5 [file Data_Sheet_1.ZIP › ImaginR_raw_data_screenshots/2-Videos_30_m/Screenshots/180/180-4.jpg]

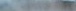

Supplement: Supplementary file 5 [file Data_Sheet_1.ZIP › ImaginR_raw_data_screenshots/2-Videos_30_m/Screenshots/180/180-1.jpg]

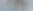

Supplement: Supplementary file 5 [file Data_Sheet_1.ZIP › ImaginR_raw_data_screenshots/2-Videos_30_m/Screenshots/180/180-2.jpg]

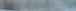

Supplement: Supplementary file 5 [file Data_Sheet_1.ZIP › ImaginR_raw_data_screenshots/2-Videos_30_m/Screenshots/180/180-9.jpg]

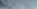

Supplement: Supplementary file 5 [file Data_Sheet_1.ZIP › ImaginR_raw_data_screenshots/2-Videos_30_m/Screenshots/180/180-8.jpg]

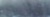

Supplement: Supplementary file 5 [file Data_Sheet_1.ZIP › ImaginR_raw_data_screenshots/2-Videos_30_m/Screenshots/613/613-10.jpg]

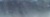

Supplement: Supplementary file 5 [file Data_Sheet_1.ZIP › ImaginR_raw_data_screenshots/2-Videos_30_m/Screenshots/613/613-1.jpg]

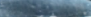

Supplement: Supplementary file 5 [file Data_Sheet_1.ZIP › ImaginR_raw_data_screenshots/2-Videos_30_m/Screenshots/613/613-3.jpg]

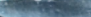

Supplement: Supplementary file 5 [file Data_Sheet_1.ZIP › ImaginR_raw_data_screenshots/2-Videos_30_m/Screenshots/613/613-6.jpg]

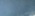

Supplement: Supplementary file 5 [file Data_Sheet_1.ZIP › ImaginR_raw_data_screenshots/2-Videos_30_m/Screenshots/613/613-4.jpg]

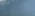

Supplement: Supplementary file 5 [file Data_Sheet_1.ZIP › ImaginR_raw_data_screenshots/2-Videos_30_m/Screenshots/613/613-9.jpg]

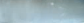

Supplement: Supplementary file 5 [file Data_Sheet_1.ZIP › ImaginR_raw_data_screenshots/2-Videos_30_m/Screenshots/002/002-6.jpg]

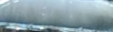

Supplement: Supplementary file 5 [file Data_Sheet_1.ZIP › ImaginR_raw_data_screenshots/2-Videos_30_m/Screenshots/002/002-7.jpg]

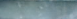

Supplement: Supplementary file 5 [file Data_Sheet_1.ZIP › ImaginR_raw_data_screenshots/2-Videos_30_m/Screenshots/002/002-5.jpg]

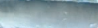

Supplement: Supplementary file 5 [file Data_Sheet_1.ZIP › ImaginR_raw_data_screenshots/2-Videos_30_m/Screenshots/002/002-4.jpg]

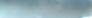

Supplement: Supplementary file 5 [file Data_Sheet_1.ZIP › ImaginR_raw_data_screenshots/2-Videos_30_m/Screenshots/002/002-1.jpg]

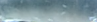

Supplement: Supplementary file 5 [file Data_Sheet_1.ZIP › ImaginR_raw_data_screenshots/2-Videos_30_m/Screenshots/002/002-3.jpg]

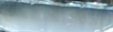

Supplement: Supplementary file 5 [file Data_Sheet_1.ZIP › ImaginR_raw_data_screenshots/2-Videos_30_m/Screenshots/002/002-2.jpg]

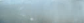

Supplement: Supplementary file 5 [file Data_Sheet_1.ZIP › ImaginR_raw_data_screenshots/2-Videos_30_m/Screenshots/002/002-10.jpg]

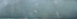

Supplement: Supplementary file 5 [file Data_Sheet_1.ZIP › ImaginR_raw_data_screenshots/2-Videos_30_m/Screenshots/002/002-9.jpg]

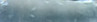

Supplement: Supplementary file 5 [file Data_Sheet_1.ZIP › ImaginR_raw_data_screenshots/2-Videos_30_m/Screenshots/002/002-8.jpg]

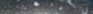

Supplement: Supplementary file 5 [file Data_Sheet_1.ZIP › ImaginR_raw_data_screenshots/2-Videos_30_m/Screenshots/183/183-7.jpg]

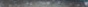

Supplement: Supplementary file 5 [file Data_Sheet_1.ZIP › ImaginR_raw_data_screenshots/2-Videos_30_m/Screenshots/183/183-6.jpg]

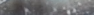

Supplement: Supplementary file 5 [file Data_Sheet_1.ZIP › ImaginR_raw_data_screenshots/2-Videos_30_m/Screenshots/183/183-4.jpg]

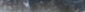

Supplement: Supplementary file 5 [file Data_Sheet_1.ZIP › ImaginR_raw_data_screenshots/2-Videos_30_m/Screenshots/183/183-5.jpg]

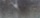

Supplement: Supplementary file 5 [file Data_Sheet_1.ZIP › ImaginR_raw_data_screenshots/2-Videos_30_m/Screenshots/183/183-1.jpg]

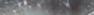

Supplement: Supplementary file 5 [file Data_Sheet_1.ZIP › ImaginR_raw_data_screenshots/2-Videos_30_m/Screenshots/183/183-2.jpg]
